# Characterization of Stimulatory Action on Voltage-Gated Na^+^ Currents Caused by Omecamtiv Mecarbil, Known to Be a Myosin Activator

**DOI:** 10.3390/biomedicines11051351

**Published:** 2023-05-03

**Authors:** Chih-Yu Ting, Chia-Lung Shih, Meng-Cheng Yu, Chao-Liang Wu, Sheng-Nan Wu

**Affiliations:** 1 Department of Emergency Medicine, Ditmanson Medical Foundation Chia-Yi Christian Hospital, Chiayi City 60002, Taiwan; 2 Clinical Research Center, Ditmanson Medical Foundation Chia-Yi Christian Hospital, Chiayi City 60002, Taiwan; 3 Department of Physiology, National Cheng Kung University Medical College, Tainan 70101, Taiwan; 4 School of Medicine, National Sun Yat-Sen University College of Medicine, Kaohsiung 80424, Taiwan

**Keywords:** myosin activator, omecamtiv mecarbil, voltage-gated Na^+^ current, transient Na^+^ current, late Na^+^ current, window Na^+^ current, cumulative inactivation, slow inactivation

## Abstract

Omecamtiv mecarbil (OM, CK-1827452) is recognized as an activator of myosin and has been demonstrated to be beneficial for the treatment of systolic heart failure. However, the mechanisms by which this compound interacts with ionic currents in electrically excitable cells remain largely unknown. The objective of this study was to investigate the effects of OM on ionic currents in GH3 pituitary cells and Neuro-2a neuroblastoma cells. In GH_3_ cells, whole-cell current recordings showed that the addition of OM had different potencies in stimulating the transient (*I*_Na(T)_) and late components (*I*_Na(L)_) of the voltage-gated Na^+^ current (*I*_Na_) with different potencies in GH_3_ cells. The EC_50_ value required to observe the stimulatory effect of this compound on *I*_Na(T)_ or *I*_Na(L)_ in GH_3_ cells was found to be 15.8 and 2.3 µM, respectively. Exposure to OM did not affect the current versus voltage relationship of *I*_Na(T)_. However, the steady-state inactivation curve of the current was observed to shift towards a depolarized potential of approximately 11 mV, with no changes in the slope factor of the curve. The addition of OM resulted in an increase in the decaying time constant during the cumulative inhibition of *I*_Na(T)_ in response to pulse-train depolarizing stimuli. Furthermore, the presence of OM led to a shortening of the recovery time constant in the slow inactivation of *I*_Na(T)_. Adding OM also resulted in an augmentation of the strength of the window Na^+^ current, which was evoked by a short ascending ramp voltage. However, the OM exposure had little to no effect on the magnitude of L-type Ca^2+^ currents in GH_3_ cells. On the other hand, the delayed-rectifier K^+^ currents in GH_3_ cells were observed to be mildly suppressed in its presence. Neuro-2a cells also showed a susceptibility to the differential stimulation of *I*_Na(T)_ or *I*_Na(L)_ upon the addition of OM. Molecular analysis revealed potential interactions between the OM molecule and hNa_V_1.7 channels. Overall, the direct stimulation of *I*_Na(T)_ and *I*_Na(L)_ by OM is assumed to not be mediated by an interaction with myosin, and this has potential implications for its pharmacological or therapeutic actions occurring in vivo.

## 1. Introduction

Omecamtiv mecarbil (OM), previously known as CK-1827452, belongs to a new class of myotropes and has been shown to augment cardiac contractility by its binding to cardiac myosin. This small molecule increases the number of force generators, or myosin heads, that interact with the actin filament and initiate a power stroke at the beginning of systole, making it the first drug to work though this mechanism [1,2,3,4]. The short-term intravenous administration of OM has been shown to improve cardiac performance in several clinical studies [5,6,7,8]. Consequently, this compound is increasingly being recognized as a beneficial treatment for acute or chronic heart failure [5,9,10]. While the myosin molecule has been previously reported to be expressed in pituitary lactotrophs [11], it remains uncertain whether or how exposure to OM is capable of perturbing the functional activities of different excitable cells.

Voltage-gated Na^+^ (Na_V_) channels are found in various excitable tissues of mammals including the central and peripheral nervous systems, as well as the endocrine and neuroendocrine systems. These channels are composed of nine isoforms (Na_V_1.1-1.9, also referred to as SCN1A-SCN5A and SCN8A-SCN11A) [12,13]. When activated, these channels can rapidly depolarize the surface membrane, resulting in the upstroke of an action potential. This intrinsic modulation of firing action potentials can influence the magnitude, frequency, and discharge pattern in different types of excitable cells [12,14]. Notably, modulators that increase the *I*_Na_ magnitude can be beneficial for the treatment of heart failure [15,16,17,18,19]. While OM is increasingly used in patients with heart failure [1,6,9,20], its specific mechanism of action on ionic currents in electrically excitable cells is not yet fully understood. Further research is necessary to fully understand the effects of OM on these channels.

The objective of this study was to investigate the direct effects of OM on membrane ionic currents in pituitary GH_3_ cells and neuroblastoma Neuro-2a cells using whole-cell patch-clamp current recordings, given the aforementioned considerations. Our findings are particularly significant, as we have obtained substantial evidence indicating that OM not only binds to myosin molecules but also enhances *I*_Na_ in a time-, state-, and concentration-dependent fashion at micromolar concentrations when exposed to cells. Moreover, our results suggest that the stimulatory effects on *I*_Na(T)_ or *I*_Na(L)_ are not mediated by OM’s binding to myosin. These findings have notable implications for the potential pharmacological, therapeutic, and adverse effects of OM in vivo.

## 2. Materials and Methods

### 2.1. Chemicals, Drugs, Reagents, and Solution Used in This Work

Omecamtiv mecarbil (OM, CK-1827452, methyl 4-(2-fluoro-3-(3-(6-Methylpyridin-3-yl)ureido)benzyl)piperazine-1-carboxylate, methyl 4-[[2-fluoro-3-[(6-methylpyridin-3-yl)carbamoylamino]phenyl]methyl]piperazine-1-carboxylate, C_20_H_24_FN_5_O_3_) was acquired from MedChemExpress (Genechain, Kaohsiung, Taiwan), while nifedipine (Nif), tetraethylammonium chloride (TEA), and tetrodotoxin (TTX) were supplied by Sigma-Aldrich (Merck, Taipei, Taiwan). The majority of the cell culture materials, such as Ham’s F-12 medium, Dulbecco’s modified Eagle’s medium, fetal bovine or calf serum, horse serum, L-glutamine, and trypsin-EDTA, were obtained from HyClone^TM^ (Thermo Fischer, Genechain). All the other chemicals and reagents used in this study, such as CsCl, CsOH, CdCl_2_, and HEPES, were of analytical grade and were obtained from commercial sources unless stated otherwise.

The study employed HEPES-buffered normal Tyrode’s solution with the following ionic composition (in mM): NaCl 136.5, CaCl_2_ 1.8, KCl 5.4, MgCl_2_ 0.53, glucose 5.5, and HEPES-NaOH buffer 5.5 (pH 7.4) as the external solution. To isolate the delayed-rectifier K^+^ current (*I*_K(DR)_), cells were immersed in Ca^2+^-free Tyrode’s solution to prevent contamination from the magnitude of Ca^2+^-dependent K^+^ currents and voltage-gated Ca^2+^ currents. The recording electrode was filled with an internal solution consisting of (in mM): K-aspartate 130, KCl 20, KH_2_PO_4_ 1, MgCl_2_ 1, Na_2_ATP 3, Na_2_GTP 0.1, EGTA 0.1, and HEPES-KOH 5 (pH 7.2) to record *I*_K(DR)_. However, to measure voltage-gated Na^+^ or Ca^2+^ currents, we replaced K^+^ ions inside the pipette internal solution with equimolar Cs^+^ ions, and the pH was adjusted to 7.2 by adding CsOH. All solutions used in this study were prepared using demineralized water obtained from a Milli-Q purification system (Merck). The filling or bathing solution and culture media were filtered using Acrodisc^®^ syringe filters with a Supor^®^ membrane with a pore size of 0.2 µm (Genechain).

### 2.2. Cell Preparations

The GH_3_ (BCRC-60015) and Neuro-2a (BCRC-60026) clonal cell strains usied in this study were obtained from the Bioresource Collection and Research Center (Hsinchu, Taiwan). GH_3_ cells were cultured in Ham’s F-12 medium supplemented with 2.5% (*v*/*v*) fetal calf serum, 15% (*v*/*v*) horse serum, and 2 mM L-glutamine. Neuro-2a cells were cultured in Dulbecco’s modified Eagle’s medium with 10% (*v*/*v*) fetal bovine serum. Both cell lines were maintained as monolayer cultures at approximately 1 × 10^6^/mL density and incubated in a humidified atmosphere containing 5% CO_2_ and 95% air at 37 °C. The cells were subcultured using trypsinization, which involved treatment with 0.025% trypsin solution (HyClone^TM^) containing 0.01% N, N-diethyldithiocarbamate, and EDTA. Electrophysiological measurements were conducted five or six days after the cells were cultured and reached 60–80% confluence.

### 2.3. Electrophysiological Measurements with the Patch-Clamp Technique

Before performing the experiments, GH_3_ or Neuro-2a cells were dispersed using 1% trypsin-EDTA solution, and a small amount of the resulting cell suspension was promptly transferred into a recording chamber which was custom-built for this study. The recording chamber was mounted on the stage of an inverted DM-IL microscope (Leica; Major Instruments, Tainan, Taiwan). The cells were then allowed to settle onto the bottom of the chamber in normal Tyrode’s solution at room temperature (20–25 °C).

The pipettes used in this study were made from Kimax^®^-51 glass tubing with a 1.5–1.8 mm outer diameter (#34500; Kimble^®^, Dogger, New Taipei City, Taiwan) using a vertical two-stage puller (PP-83; Narishige, Taiwan Instrument, Tainan, Taiwan). The tips of the pipettes were fire-polished using an MF-83 microforge (Narishige). The electrode used for the measurements typically had a tip resistance of 3–5 MΩ when filled with different internal solutions, as stated above. We measured different types of ionic currents in the whole-cell configuration using a modified patch-clamp technique with either an Axoclamp-2B (Molecular Devices, Sunnyvale, CA) or an RK-400 amplifier (Bio-Logic, Claix, France), as previously described [21,22,23]. We obtained GΩ-seals in an all-or-nothing fashion, resulting in a significant improvement in the signal-to-noise ratio. We zeroed the liquid junction potentials, which can arise when ionic compositions of the bath solution and pipette internal solution differ, shortly before achieving GΩ-seal. We then corrected the whole-cell data, as previously described [21].

### 2.4. Data Recordings and Analyses

The signal output, including the membrane potential and current tracings, was continuously monitored and digitally acquired at a sampling frequency of 10 kHz or higher, using an ASUS ExpertBook laptop computer (Yuan-Dai, Tainan, Taiwan). To facilitate effective analog-to-digital (A/D) and digital-to-analog (D/A) conversion, we connected a Digidata^®^-1440A interface to the laptop computer via a USB 2.0 port. We utilized the pClamp 10.6 software, implemented under Microsoft Windows 7 (Redmond, WA), to control the interface. Our voltage-clamp protocols consisted of variable rectangular and ramp waveforms, which were digitally created and imposed on the tested cells through D/A conversion. We utilized a dual output pulse stimulator (Astro-Med Grass S88X; Grass, West Warwick, RI) to provide pulse-train (PT) stimulation.

**Figure 1:**
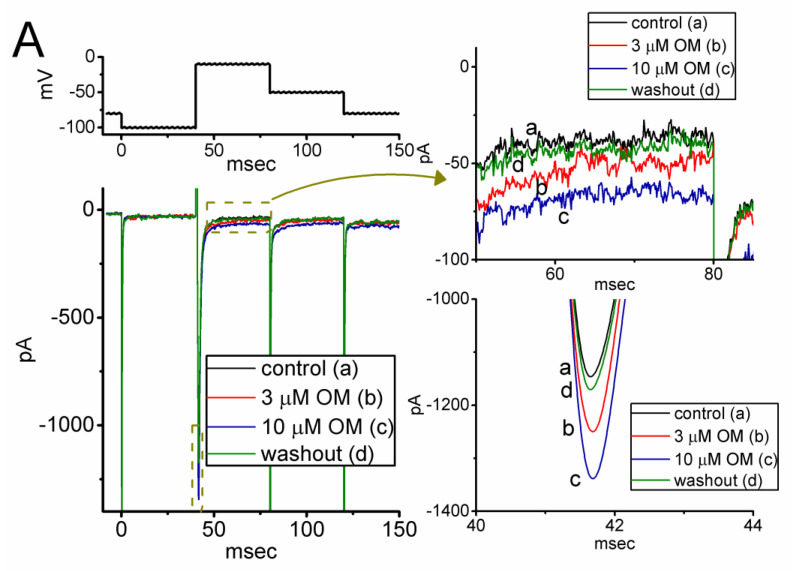

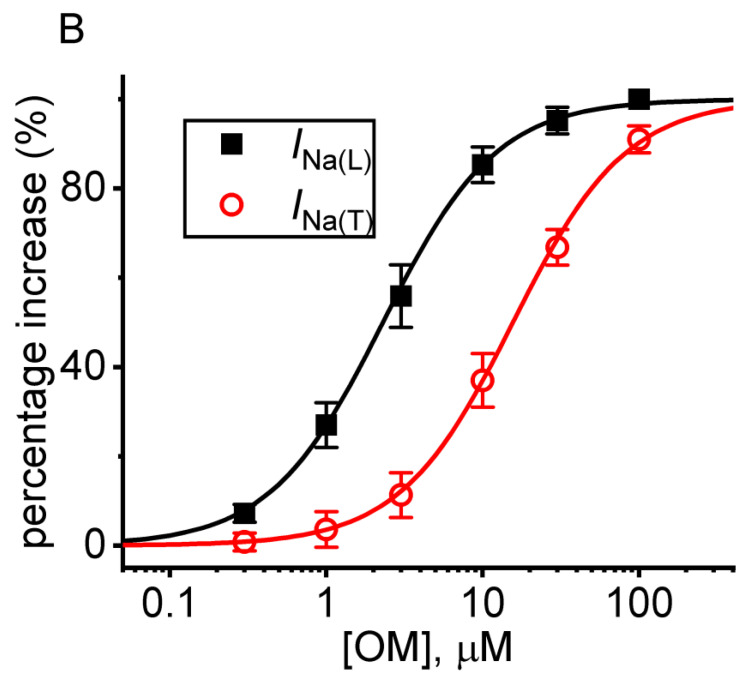
Stimulatory effect of OM on voltage-gated Na^+^ current (*I*_Na_) measured from pituitary GH_3_ cells. This series of experiments was conducted in ells placed in Ca^2+^-free, Tyrode’s solution which contained 10 mM tetraethylammonium chloride (TEA) and 0.5 mM CdCl_2_, and we filled up the electrode with Cs^+^-containing solution. (**A**) Representative current traces achieved in the control period (a, the absence of OM), with the presence of 3 µM OM (b) or 10 µM OM (c), and washout of OM (d). The voltage pulse protocol given is shown in the upper part. The right side in (**A**) demonstrates the expanded records from each broken box with curve arrow. (**B**) Concentration-dependent relationship of OM (0.3−100 µM) on the transient (*I*_Na(T)_, red open circles) and late (*I*_Na(L)_, black filled squares) components of *I*_Na_ evoked by short membrane depolarization (mean ± SEM; n = 8 for each red or black point). Each sigmoidal curve indicated in black or red color represents the best fit to the Hill equation, as described in detail in the Materials and Methods section.

To evaluate the percentage increase in OM on both the transient (*I*_Na(T)_) and late component (*I*_Na(L)_) of *I*_Na_ (as demonstrated in Figure 1B), GH_3_ cells were placed in Ca^2+^-free, Tyrode’s solution, each cell was stepped from −100 to −10 mV for a duration of 30 ms, and the amplitudes of *I*_Na(T)_ and *I*_Na(L)_ during the exposure to different OM concentrations were measured. The *I*_Na(T)_ or *I*_Na(L)_ amplitude was measured at the beginning or end of the short depolarization step, respectively. The *I*_Na(L)_ amplitudes in the presence of 100 µM were taken as 100%, and the *I*_Na(T)_ or *I*_Na(L)_ amplitudes were collected and then compared with those obtained following the addition of different OM concentrations (0.3–100 µM). The concentration–response relationships for the OM-induced stimulation of *I*_Na(T)_ or *I*_Na(L)_ observed in GH_3_ cells were optimally determined by the Hill equation with a nonlinear regression analysis:

percentage increase%=Emax×[OM]nHEC50nH+[OM]nH

where [OM] is the OM concentration given, EC_50_ and n_H_ represent the half-maximal concentration of OM and the Hill coefficient, respectively, and E_max_ is the maximal activation of *I*_Na(T)_ or *I*_Na(L)_ caused by the OM presence.

To determine the quasi-steady-state inactivation curve of *I*_Na(T)_ in the presence or absence of 10 µM OM, we employed a two-step voltage-clamp protocol on the cells under examination. The measurements involved applying a 30 ms conditioning potential to a series of voltages ranging from −100 to 0 mV, following a holding potential of −100 mV, before a 30 ms test pulse from −80 to −10 mV from a holding potential of −100 mV was applied to precede a 30 ms test pulse from −80 to −10 mV. The time interval between the two sets of voltage pulses was approximately 2 min to ensure the complete recovery of *I*_Na_ evoked by the rapid depolarizing step. We constructed the relationships between the conditioning potentials and the normalized amplitude (I/I_max_) with the absence or presence of 10 µM OM by fitting the dataset to a modified Boltzmann function—that is,

I=Imax1+exp(V−V12)/k

where *I*_max_ is the maximal amplitude of *I*_Na_ in the absence and presence of 10 µM OM; *V* and *V*_1/2_ represent the membrane potential in mV and the half-point of the inactivation curve of the current, respectively; and *k* is the slope factor of the curve.

### 2.5. Curve-Fitting Methods and Statistical Analyses Used in This Work

The experimental datasets were fitted with either linear or nonlinear, such as exponential, or sigmoidal curves. We used the interactive least-squares procedure to perform the curve fitting, utilizing tools such as Microsoft Excel^®^’s embedded “Solver” (Redmond, WA) or OriginPro^®^ 2021 (OriginLab; Scientific Formosa, Kaohsiung, Taiwan). We present the results as the mean values with the standard error of the mean (SEM), and we report the sample sizes (i.e., cell number).

For comparisons between two groups, paired or unpaired *t*-tests were performed. For comparisons among more than two groups, analysis of variance (ANOVA-1 or ANOVA-2) was used, with or without repeated measures. Post-hoc Fisher’s least-significant difference method was then applied to determine the differences between groups. Statistical analyses were mostly made using the SPSS 20 software (AsiaAnalytics, Taipei, Taiwan). Statistical significance (indicated with * or ** in the figures) was determined at a *p*-value of <0.05.

## 3. Results

### 3.1. Effect of OM on Voltage-Gated Na^+^ Current (I_Na_) in Pituitary GH_3_ Cells

During the first stage of whole-cell current recordings, we measured the effects of OM on the magnitude and gating properties of *I*_Na_ in response to a rapid depolarization step. The cells were immersed in a Ca^2+^-free, Tyrode’s solution containing 10 mM tetraethylammonium chloride (TEA), and the measuring pipette was filled with a Cs^+^-enriched internal solution. As shown in Figure 1A, one minute after continuous exposure to OM at a concentration of 3 or 10 µM, we observed a progressive increase in the amplitudes of both the transient (*I*_Na(T)_) and late components (*I*_Na(L)_) of *I*_Na_ in response to a short depolarizing pulse from −100 to −10 mV for a duration of 40 ms. For example, as the rectangular voltage step from −100 to −10 mV for a duration of 40 ms was given (indicated in the upper part of Figure 1A), the application of OM at a concentration of 3 or 10 µM was noticed to result in an evident increase in *I*_Na(T)_ and *I*_Na(L)_ amplitudes, respectively, to 1245 ± 32 pA (n = 8, *p* < 0.05) and 49 ± 6 pA (n = 8, *p* < 0.05) (in the presence of 3 µM OM) or to 1326 ± 39 pA (n = 8, *p* < 0.05) and 77 ± 8 pA (n = 8, *p* < 0.05) (in the presence of 10 µM OM), from control values of 1092 ± 26 pA (n = 8) and 31 ± 4 pA (n = 8). After the removal of OM, the *I*_Na(T)_ and *I*_Na(L)_ amplitudes were returned to 1102 ± 27 pA and 32 ± 5 pA, respectively. Furthermore, we exposed GH_3_ cells to 1 µM tetrodotoxin (TTX) alone, and the *I*_Na(T)_ amplitude evoked by the same voltage protocol was almost abolished, as evidenced by a significant reduction in the current amplitude to 42 ± 4 pA (n = 7, *p* < 0.01) from a control value of 1089 ± 27 pA (n = 7).

Figure 1B demonstrates that the application of OM to the bath causes a concentration-dependent increase in the amplitude of *I*_Na)T)_ (red open circles) and *I*_Na(L)_ (black filled squares) evoked in response to the short depolarizing pulse from −100 to −10 mV. The EC_50_ values tailored for the OM-stimulated *I*_Na)T)_ and *I*_Na(L)_ amplitude seen in GH_3_ cells were calculated using the Hill equation described in the Materials and Methods section, and they were found to be 15.8 and 2.3 µM, respectively. These values were significantly different between its stimulatory effects on these two components of the current. Our initial measurements indicate that OM has a preferential stimulatory effect on the natively expressed *I*_Na(L)_ over the *I*_Na(T)_ elicited by the rectangular depolarization step in GH_3_ cells.

### 3.2. The Steady-State Current versus Voltage (I–V) Relationship and the Inactivation Curve of I_Na(T)_ Modified by the OM Presence

**Figure 2:**
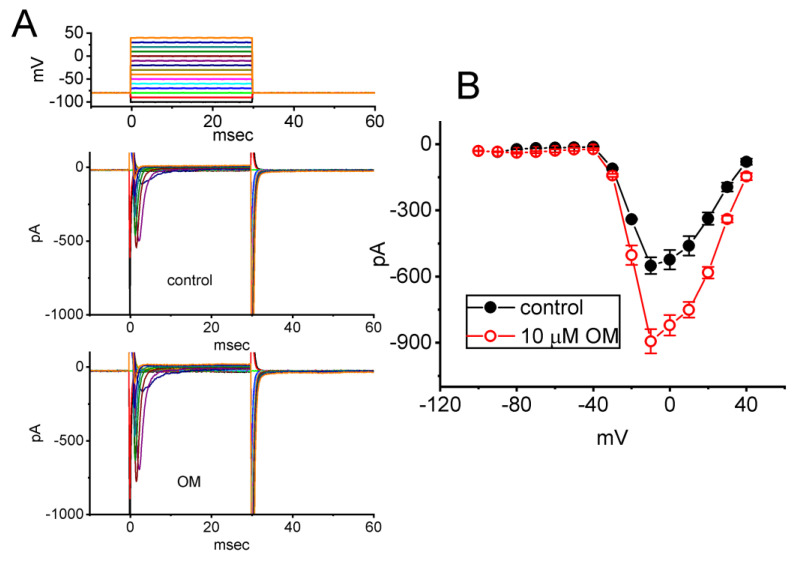

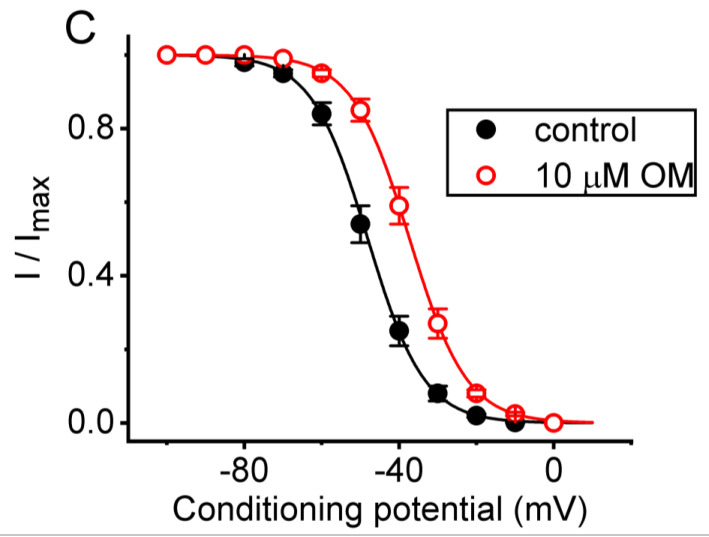
Effect of OM on the current versus voltage (*I*–*V*) relationship (**A**) and the steady-state inactivation curve (**B**) of *I*_Na(T)_ identified from GH_3_ cells. (**A**) Representative current traces acquired from the absence (upper) and the presence (lower) of 10 µM OM. The uppermost part shows the voltage pulse protocol applied. In (**B**), the mean *I*–*V* curves of the current were obtained in the absence (black filled circles) and presence (red open circles) of 10 µM OM (mean ± SEM; n = 8 for each red or black point). (**C**) Quasi−steady−state inactivation curve of *I*_Na(T)_ taken with (red open circles) or without the addition (black filled circles) of 10 µM OM. The two−step voltage protocol was applied to evoke the current, and each point shown in the graph represents the mean ± SEM (n = 8). The sigmoidal curves were obtained with the least-squares fit to a Boltzmann function, as elaborated on in Materials and Methods.

To investigate the stimulatory effect of OM on *I*_Na_, we examined whether this compound affected the steady *I*–*V* relationship of *I*_Na_ in GH_3_ cells. For these measurements, cells were clamped at −80 mV, and a series of voltages ranging from −100 to +40 mV in 10-mV steps for a duration of 30 ms was applied to evoke *I*_Na_. We then assessed whether OM exerted any perturbations on the *I*–*V* relationship of *I*_Na_. Figure 2A shows a representative current trace at different levels of membrane potentials, and Figure 2B depicts the mean *I*–*V* relationships of *I*_Na(T)_ obtained with or without exposure to 10 µM OM. The results indicate that no obvious change in the overall steady-state *I*–*V* relationship of *I*_Na(T)_ was observed during cell exposure to OM.

We further characterized the effect of OM on the inactivation curve of *I*_Na(T)_, specifically examining the relationship of the normalized amplitude and the conditioning potential. Figure 2C illustrates the steady-state inactivation curve of the current in the absence of OM and during exposure to 10 µM OM. The sigmoidal curve derived from the datasets was well fitted with the modified Boltzmann equation (stated under Materials and Methods). That is, the values of *V*_1/2_ and *k* obtained in the control period (i.e., OM was not present) were −48.1 ± 2.2 mV (n = 8) and 7.3 ± 0.1 (n = 8), respectively, while those during exposure to 10 µM OM were −37.3 ± 2.1 mV (n = 8, *p* < 0.05) and 7.4 ± 0.1 (n = 8, *p* > 0.05). These results enable us to indicate that the presence of 10 µM OM could cause a rightward shift along the voltage axis in the steady-state inactivation curve by approximately 11 mV. However, the slope factor of the curve did not show a significant difference between the absence and presence of 10 µM OM. This indicates that the gating charge of the curve did not change in the presence of OM.

### 3.3. OM-Induced Retardation in the Cumulative Inhibition of I_Na(T)_ Inactivation during Pulse-Train (PT) Depolarizing Stimuli

**Figure 3:**
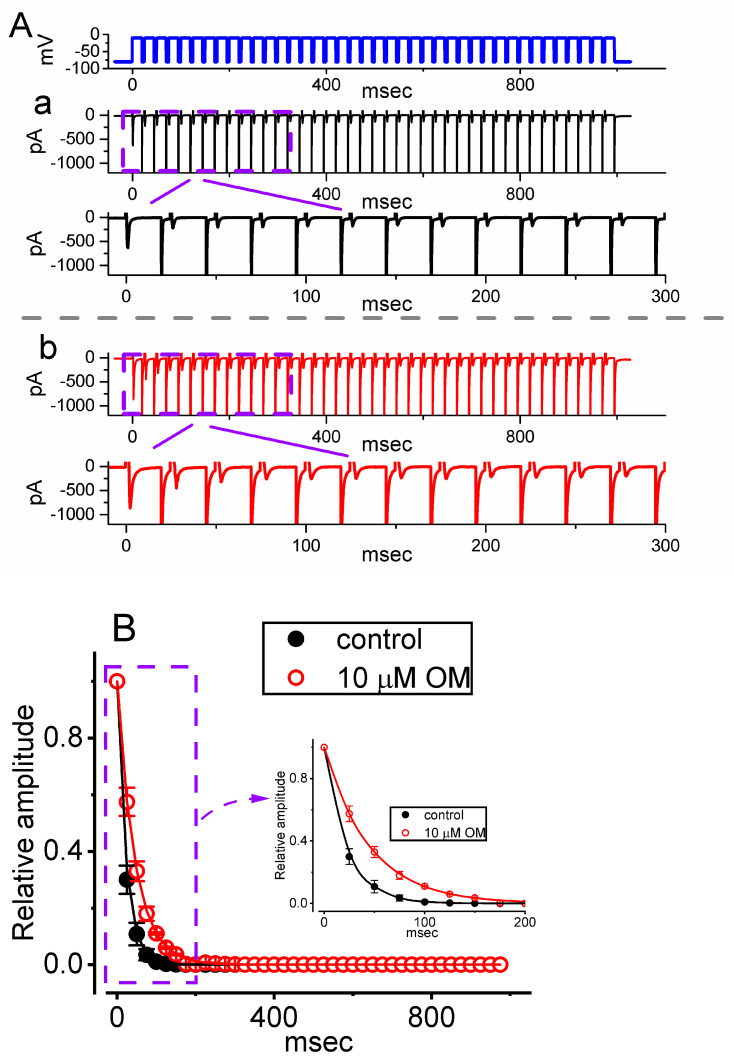
Effect of OM on *I*_Na(T)_ evoked by a pulse train (PT) of depolarizing pulses in GH_3_ cells. The voltage protocol (indicated in the uppermost part) employed a consistency of 40 20 ms pulses (stepped to −10 mV) separated by a 5 ms interval at −80 mV for a total duration of 1 s. (**A**) Representative current traces taken during the control period (a, absence of OM) or with cell exposure to 10 µM OM (b). To demonstrate a single *I*_Na_ trace, the lower part in each panel (a, b) indicates the expanded record from the purple, broken box of the upper part. (**B**) The relationship of the relative *I*_Na(T)_ versus the PT duration obtained without (black filled circles) or with (red open circles) the application of 10 µM OM (mean ± SEM; n = 8 for each red or black point). The inset displays a magnified view of the record within a purple dashed box, indicated by a curved arrow. Each continuous line drawn was approximately fitted by a single exponential. Notably, cell exposure to 10 µM OM caused a slowdown of the time course of *I*_Na(T)_ inactivation elicited by a PT depolarizing stimulation from −80 to −10 mV for 1 s, as well as an increase in the time constant of *I*_Na_ inactivation during the PT depolarizing pulse.

Previous studies have shown that the inactivation of *I*_Na(T)_ can accumulate over time prior to activation during PT short pulses [22,24,25]. Additionally, the effect of OM on cardiac contractility has been reported to be rate-dependent [26]. To investigate whether the presence of OM could modify the inactivation process of *I*_Na(T)_ during PT membrane depolarization, we performed experiments using a GH_3_ cell. The cell was clamped at −80 mV, and a stimulus protocol consisting of repetitive depolarizations to −10 mV was applied for a duration of 1 s, with each single pulse lasting 20 ms and a rate of 40 Hz (as indicated in the uppermost part of Figure 3A). As demonstrated in Figure 3, the process of *I*_Na(T)_ inactivation observed in GH_3_ cells was overly evoked by a 1 s PT depolarization from −80 to −10 mV, and an evolving decaying time constant (τ) of 22.6 ± 1.3 ms (n = 8) in the control period (i.e., absence of OM) was yielded. In other words, there appeared to be a sudden *I*_Na_ decay in a single-exponential fashion during such PT depolarizing stimuli [22,24]. Furthermore, we observed a significant increase in the decay time constant (τ) of *I*_Na(T)_ when the cells were exposed to 10 µM OM during a 1 s PT depolarizing step. The τ value of the *I*_Na(T)_ decay evoked by the same PT depolarizing voltage pulse was markedly raised to 45.2 ± 1.4 mV (n = 8, *p* < 0.05), in addition to a clear increase in the *I*_Na(T)_ amplitude (Figure 3). These findings suggest that the inactivation of *I*_Na(T)_ activated during the depolarization step, which is known to accumulate over time, was also affected by OM exposure. Specifically, the inactivation process was retarded in cells exposed to OM, in addition to the increase in the *I*_Na(T)_ amplitude. 

### 3.4. Effect of OM on the Recovery Time Course in the Slow Inactivation of I_Na(T)_ Recorded from GH_3_ Cells

**Figure 4:**
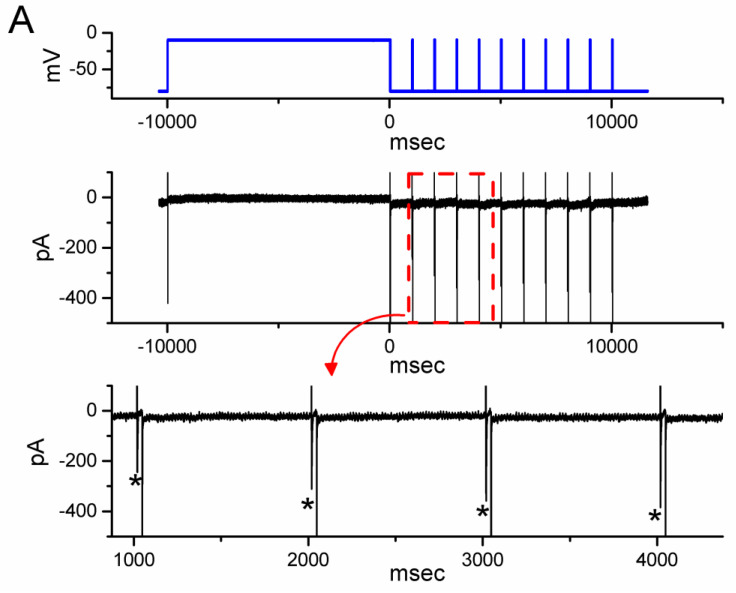

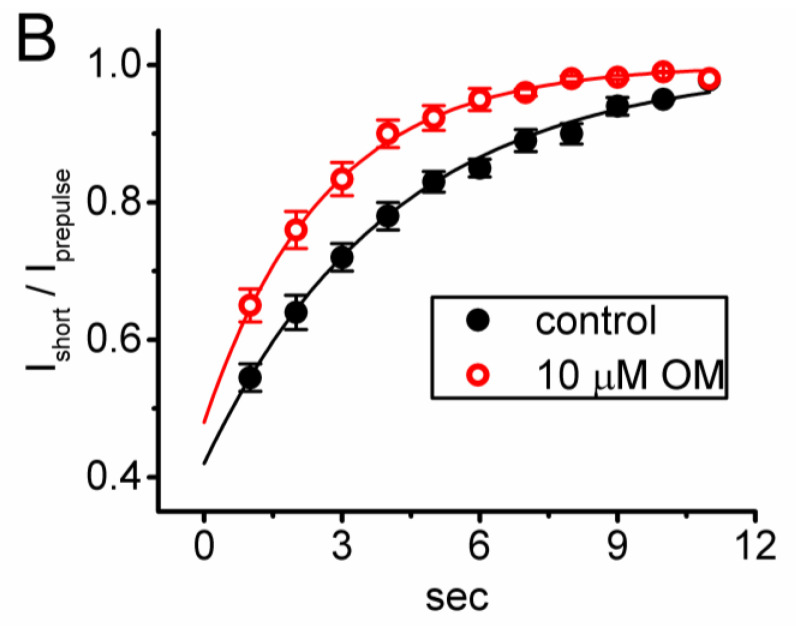
Effect of OM on the time course of recovery from *I*_Na_ inactivation activated by a depolarization step from −80 to −10 mV for a duration of 10 s (prepulse). Following the 10 s prepulse, the recovery time course of the current amplitude was consecutively measured during a 30 ms depolarizing step from −80 to −10 mV with a rate of 1 Hz. (**A**) Representative current trace obtained in the control period. The uppermost part (indicated in blue color) depicts the voltage pulse protocol given, while the lower part shows the display of an expanded record in a red broken box with a red curve arrow appearing in the upper part. The asterisks marked in the lower part indicate a progressive increase in the *I*_Na(T)_ amplitude (i.e., recovery time course). (**B**) The relationship of the relative amplitude of *I*_Na_ versus the stimulus duration achieved without (black filled circles) and with (red open circles) the presence of 10 µM OM (mean ± SEM; n = 8 for each red or black point). The relative amplitude (i.e., *I*_short_/*I*_prepulse_) appearing at the y axis was the ratio of the *I*_Na(T)_ amplitude during a short 30 ms depolarizing pulse at different times (i.e., with a stimulus rate of 1 Hz) and that activated during a 10 s prepulse from −80 to −10 mV. Notably, the presence of OM produced a clear shortening in the recovery time course of *I*_Na(T)_ inactivation taken after a 10 s prepulse depolarization. Each black asterisk on the graph represents the occurrence of a transient Na+ current.

Previous research has shown that the slow inactivation of *I*_Na(T)_ can occur during prolonged membrane depolarization in vomeronasal sensory neurons, which is thought to be closely linked to time-dependent, short-term, and spike-frequency adaptation [27]. To investigate whether OM is able to modify the recovery time course of this slow inactivation of *I*_Na(T)_, we performed further experiments. We applied a prepulse with a 10 s maintained depolarization from −80 to −10 s, and a subsequent short depolarization test step at a rate of 1 Hz was given to the tested cell [27]. As demonstrated in Figure 4, following a 10 s depolarizing prepulse, the initial *I*_Na_ was smaller in the magnitude, and with an increased number of the depolarizing pulse, the current amplitude became progressively increased in a single-exponential process. The results reflected that there was an emergence in the recovery time course of *I*_Na_ activated following the slow inactivation of the current by a prolonged depolarizing prepulse for a duration of 10 s. Furthermore, with cell exposure to 10 µM OM, the τ value of such recovery time course was markedly diminished to 2.6 ± 0.3 s (n = 8, *p* < 0.05) from a control value of 4.1 ± 0.4 s (n = 8). Based on the results, it is reasonable to speculate that the OM presence can enhance the recovery in the slow inactivation of *I*_Na(T)_ observed in these cells.

### 3.5. Stimulatory Effect of OM on the Window Component of I_Na_ (I_N(W)_) Identified from GH_3_ Cells

**Figure 5:**
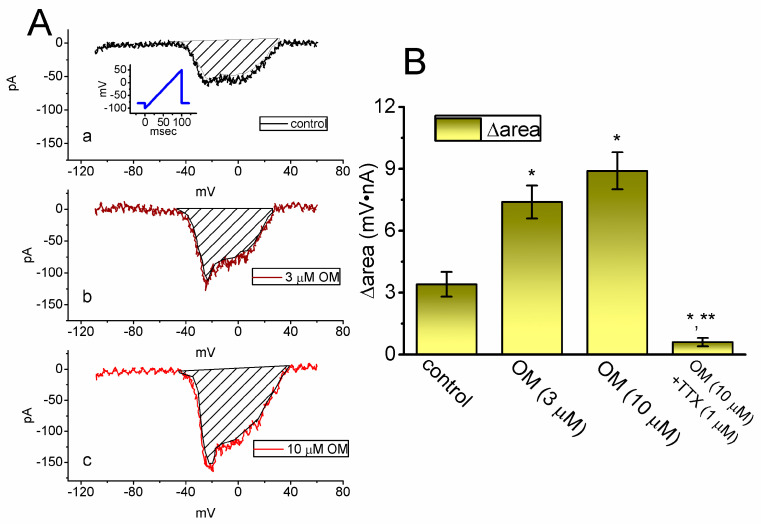
Stimulatory effect of OM on the nonlinear window Na^+^ current (*I*_Na(W)_) activated by a short ascending ramp voltage (V_ramp_) identified from GH_3_ cells. This set of whole−cell current measurements was conducted with the tested cells kept in Ca^2+^-free, Tyrode’s solution, and we clamped the cell at −80 mV and thereafter applied an ascending V_ramp_ from −100 to +50 mV for a duration of 100 ms (i.e., with a ramp speed of +1.5 mV/ms). (**A**) Representative current traces achieved in the control period (upper, a) and during cell exposure to 3 µM OM (middle, b) or 10 µM OM (lower, c). The voltage pulse protocol applied is shown in the inset of the upper part, while the shaded area in each plot represents the change in the area (Δarea) of the activated *I*_Na(W)_ current, caused by a sudden increase in the upsloping V_ramp_. (**B**) Summary bar graph demonstrating the effects of OM (3 and 10 µM) and OM (10 µM) plus tetrodotoxin (TTX, 1 µM) on the Δarea of V_ramp_-activated *I*_Na(W)_ (mean ± SEM; n = 8 for each yellow bar). The Δarea (i.e., the relationship of the membrane voltage versus the current amplitude) was calculated at the shaded area in (**A**), which is encircled under the voltage ranging between −60 and +40 mV during the short ascending V_ramp_. * Significantly different from the control (*p* < 0.05); ** significantly different from the OM (10 µM) −alone group (*p* < 0.05).

In recent studies, the instantaneous *I*_Na(W)_ current has been shown to be detectable during the short duration of an upwardly sloping (or ascending) ramp voltage (V_ramp_) in various excitable cells [23,28,29]. In this study, we investigated whether the presence of OM could alter the amplitude of the non-linear *I*_Na(W)_ that is elicited by a brief, upwardly sloping V_ramp_. To perform this separate set of experiments, we clamped the tested cell at −80 mV and applied an ascending V_ramp_ from −100 to +50 mV for a duration of 100 ms (i.e., with a ramp speed of 1.5 mV/ms) to activate the instantaneous *I*_Na(W)_. As shown in Figure 5, within one minute of exposing GH_3_ cells to 10 µM OM, the strength (i.e., Δarea) of *I*_Na(W)_ activated by the 100 ms upsloping V_ramp_ was markedly increased. For example, treating cells with OM at a concentration of 3 or 10 µM resulted in a significant increase in *I*_Na(W)_’s Δarea to 7.4 ± 0.8 mV·nA (n = 8, *p* < 0.05) or 8.9 ± 0.9 mV·nA (n = 8, *p* < 0.05), respectively, from a control value of 3.4 ± 0.6 mV·nA (n = 8). Additionally, when cells were continuously exposed to 10 mM OM, the further addition of 1 µM TTX effectively suppressed the Δarea of *I*_Na(W)_ to 0.6 ± 0.2 mV·nA (n = 8, *p* < 0.05). Based on these experimental observations, it is evident that the presence of OM can increase the strength of *I*_Na(W)_ that is activated by the ascending V_ramp_.

### 3.6. Inability of OM to Affect the Magnitude of L-Type Ca^2+^ Current (I_Ca,L_) Identified in GH_3_ Cells

**Figure 6:**
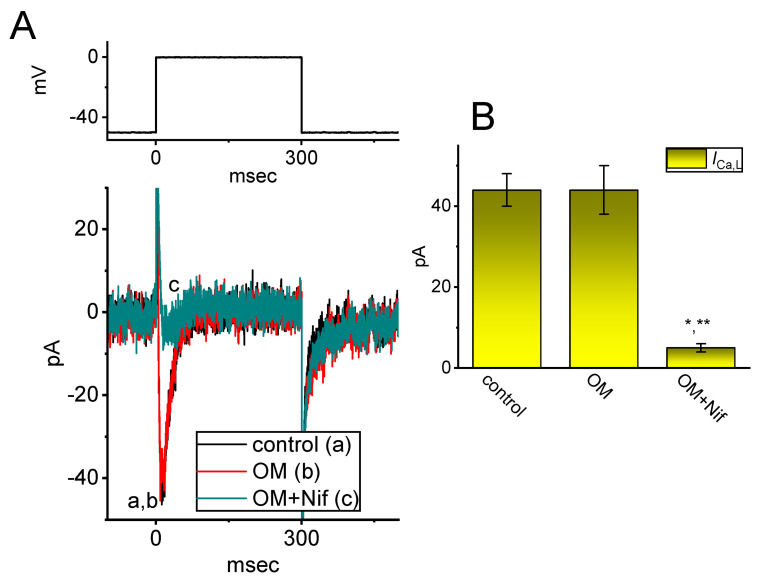
The failure of the OM effect on the L-type Ca^2+^ current (*I*_Ca,L_) identified in GH_3_ cells. These experiments were conducted in cells kept in normal Tyrode’s solution containing 1.8 mM CaCl_2_, 1 µM tetrodotoxin (TTX), and 10 mM tetraethylammonium chloride (TEA), and the recording electrode was filled up with a Cs^+^-enriched solution. (**A**) Representative *I*_Ca,L_ traces obtained in the control period (a) and with cell exposure to 10 µM OM (b) or 10 µM OM plus 1 µM nifedipine (Nif) (c). The voltage protocol given is illustrated atop current traces. (**B**) Summary bar graph demonstrating the effect of OM (10 µM) and OM (10 µM) plus Nif (1 µM) on the amplitude of *I*_Ca,L_ (mean ± SEM; n = 8 for each yellow bar). The peak amplitude of *I*_Ca,L_ was taken at the onset of the depolarizing step from −50 to 0 mV for a duration of 300 ms. * Significantly different from the control (*p* < 0.05); ** significantly different from the OM (10 µM) −alone group (*p* < 0.05).

Previous studies have reported that *I*_Ca,L_ is functionally expressed in these cells [30]. We aimed to investigate whether the presence of OM could modify the response of *I*_Ca_ to membrane depolarization. The experiments were performed in cells maintained in normal Tyrode’s solution containing 1.8 mM CaCl_2_. The measuring pipette was filled with Cs^+^-enriched solution. After one minute of exposing 10 µM OM, the amplitude of *I*_Ca,L_ activated by a 300 ms depolarization step from −50 to 0 mV remained unaltered (Figure 6). We obtained similar results in eight cells that were examined. However, with continued exposure to 10 µM OM, the addition of nifedipine (Nif, 1 µM), an inhibitor of *I*_Ca,L_ [30], effectively suppressed the *I*_Ca,L_ amplitude seen in GH_3_ cells. Therefore, unlike *I*_Na(T)_, *I*_Na(L)_, or *I*_Na(W)_ described above, the *I*_Ca,L_ in these cells was not affected by the presence of OM.

### 3.7. Mild Inhibition of Delayed-Rectifier K^+^ Current (I_K(DR)_) Produced by the OM Presence

**Figure 7:**
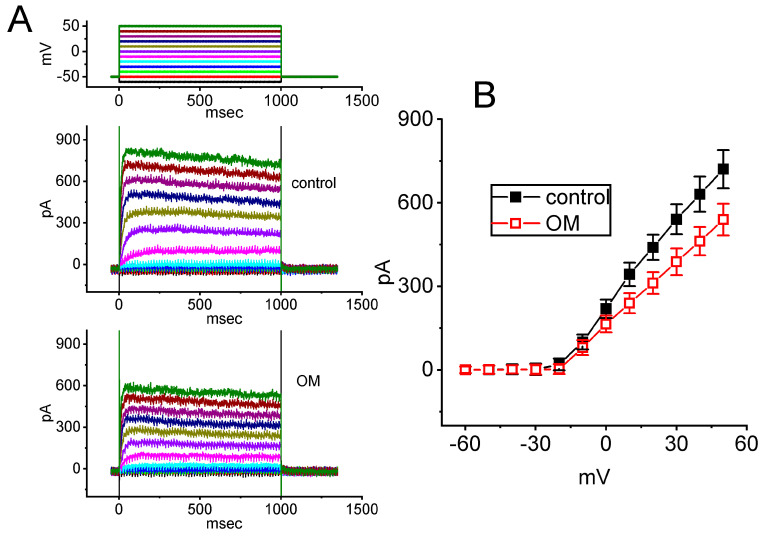
Inhibitory effect of OM on the delayed−rectifier K^+^ current (*I*_K(DR)_) recorded from GH_3_ cells. Cells were placed in Ca^2+^-free Tyrode’s solution, and the measuring pipette was filled with a K^+^-enriched solution. (**A**) Representative current traces obtained in the control period (upper) and with cell exposure to 10 µM OM (lower). The voltage protocol applied is indicated at the top. Colors in potential traces correspond to current traces evoked by different levels of voltage commands. (**B**) Mean *I*–*V* relationship of *I*_K(DR)_ obtained with and without the application of 10 µM OM (mean ± SEM; n = 7 for each point). The *I*_K(DR)_ amplitude was obtained at the end of each depolarizing pulse with the absence (black filled squares) and presence (red open circles) of 10 µM OM.

Another set of measurements was conducted to determine whether exposure to OM could alter the magnitude of K^+^ currents, specifically *I*_K(DR)_, in GH_3_ cells. The cells were maintained in Ca^2+^-free, Tyrode’s solution containing 1 µM TTX and 0.5 mM CdCl_2_, while the recording electrode was filled with a K^+^-containing solution. The mean *I*–*V* relationship of *I*_K(DR)_ was then recorded in the presence and absence of OM, and the results are presented in Figure 7. The presence of 10 µM OM was noticed to produce a slight reduction in the *I*_K(DR)_ amplitude. For example, with cell exposure to 10 µM OM, the *I*_K(DR)_ amplitude measured at the level of +50 mV was significantly decreased to 540 ± 57 pA (n = 7, *p* < 0.05) from a control value of 721 ± 69 pA (n = 7), while for those at 0 mV, the *I*_K(DR)_ amplitude was reduced from 219 ± 33 pA (n = 7) to 164 ± 30 pA (n = 7, *p* < 0.05) upon cell exposure to 10 µM OM. After the washout of the compound, the current amplitude was returned to 718 ± 65 pA (n = 7). Conversely, no measurable change in the inactivation time course of *I*_K(DR)_ was observed by adding OM. The data suggest that cell exposure to OM leads to a mild suppression of the *I*_K(DR)_ present in GH_3_ cells.

### 3.8. Effect of OM on Depolarization-Evoked I_Na_ Identified from Neuroblastoma Neuro-2a Cells

**Figure 8:**
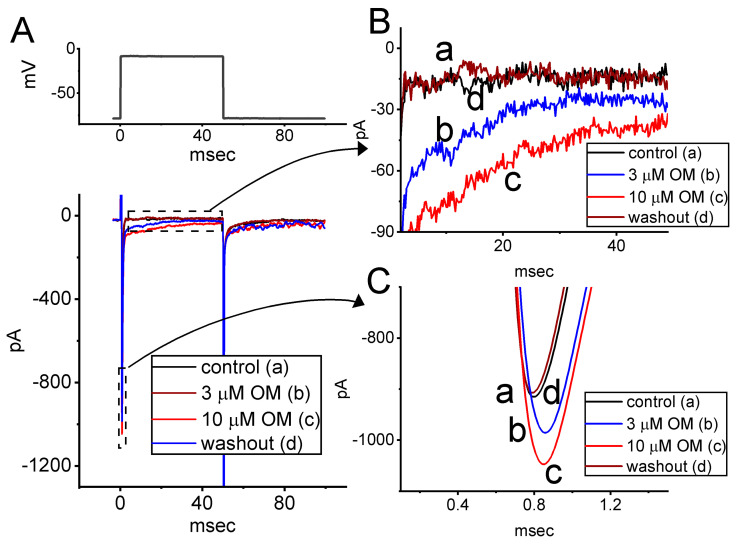
Stimulatory effect of OM on *I*_Na_ recorded from neuroblastoma Neuro-2a cells. We conducted this set of measurements in cells placed into Ca^2+^-free, Tyrode’s solution, and the electrode was then filled up with Cs^+^-enriched solution. (**A**) Representative current traces obtained in the control period (a), with cell exposure to 3 µM OM (b) or 10 µM OM (c), and washout of the compound (d). The voltage pulse protocol applied is indicated in the upper part. In (**B**,**C**), the expanded records from each broken box with a curve arrow are illustrated, respectively.

In a separate set of whole-cell experiments, we investigated whether OM could modify *I*_Na_ in another type of excitable cells—specifically, Neuro-2a cells. This cell line is widely used as a neuronal model in electrophysiology and pharmacology studies [20,31,32], and it has been shown to express Na_V_ mRNAs (Na_V_1.2, Na_V_1.3, Na_V_1.4, and Na_V_1.7) and to exhibit Na_V_channel activity [20,32]. The preparations for these cells are detailed in the Materials and Methods section. The cells were kept in Ca^2+^-free Tyrode’s solution containing 10 mM TEA, and the recording pipette was filled up with Cs^+^-enriched solution. Following continuous exposure to OM at concentrations of 3 or 10 mM for one minute, the amplitude of *I*_Na(T)_ was conceivably increased to 991 ± 22 pA (n = 7, *p* < 0.05) and 1096 ± 29 pA (n = 7, *p* < 0.05), respectively, from a control value of 792 ± 19 pA (n = 7). After the washout of OM, the amplitude of *I*_Na(T)_ returned to 802 ± 19 pA (n = 7) (Figure 8). In addition, the amplitude of *I*_Na(L)_ obtained in the presence of 3 or 10 µM OM was concurrently raised to 29 ± 6 pA (n = 7, *p* < 0.05) or 42 ± 7 pA (n = 7, *p* < 0.05), respectively, from a control value of 13 ± 4 pA (n = 7). These results were similar to those in GH_3_ cells and demonstrate that OM is capable of stimulating *I*_Na_ in response to short depolarizing steps in Neuro-2a cells.

### 3.9. Docking Prediction of hNa_V_1.7 and OM

**Figure 9:**
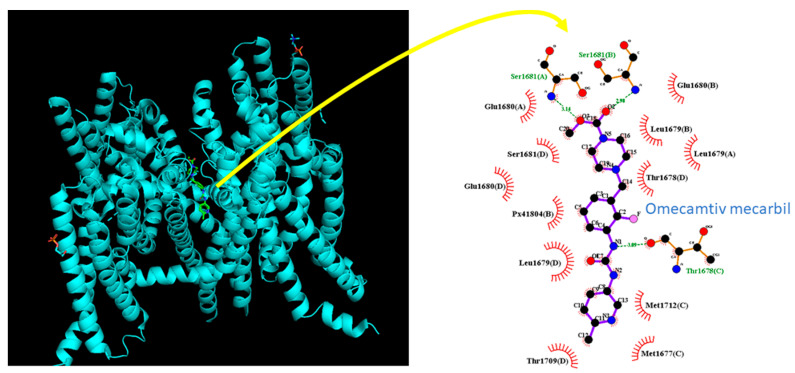
Molecular docking of the MO molecule (CID: 11689883) to the hNav1.7 channel protein (PDB ID: 5EK0). The docking prediction was generated using PyRx software (http://pyrx.sourceforge.io/) (accessed on 27 March 2023). The interaction between the hNa_V_1.7 channel and the OM molecule is depicted using LigPlot^+^ (http://www.ebi.ac.uk/thornton-sev/software/LIGPLOT/) (accessed on 27 March 2023). The hydrophobic interaction is indicated by red arcs with small bars facing and radiating toward the ligand, while green dotted lines represent the formation of hydrogen bonds between the amino-acid residues (Ser1681(A), Ser1681(B), and Thr 1678(C)) and the OM molecule.

In this study, we used molecular docking analysis with PyRx software to investigate the potential binding of OM to the hNa_V_1.7 channel protein, based on the crystal structure of hNa_V_1.7 obtained from the RCB PDB (ID: 5EK0). The predicted docking sites of OM with amino-acid residues are presented in Figure 9. Notably, the OM molecule may form hydrophobic contacts with Met1677(C), Thr1678(D), Leu1679(A), Leu1679(D), Glu1680(A), Glu1680(B), Gllu1680(D), Ser1681(D), Thr1709(D), Met1712(C), and Px41804(B). Moreover, the OM molecule was found to form hydrogen bonds with Thr1678(C), Ser1681(A), or Ser1681(B) with distances of 3.09, 3.14, or 2.98 Å.

Based on the Na_V_1.7 protein sequence (GeneBank: ASY-04966.1), we identified the inactivation gate of the channel at residue positions ranging between 1459 and 1462, which are adjacent to the OM docking sites. Our molecular docking results suggest that OM can dock to the transmembrane segment (position: 1665–1683) of the hNa_V_1.7 channel (PDB: 5EK0) with a binding affinity estimated as −9.5 kcal/mol. Combining these results with the electrophysiological data, we propose that OM can have a significant impact on the magnitude and/or gating kinetics of *I*_Na_.

## 4. Discussion

The major findings presented in this study are as follows: (i) GH_3_-cell exposure to OM produced a concentration-dependent increase in transient (*I*_Na(T)_) and late components (*I*_Na(L)_) of *I*_Na_, with effective EC_50_ values of 15.8 and 2.3 µM, respectively; (ii) the *I*–*V* relationship of *I*_Na(T)_ was unaffected with the presence of OM; however, the steady-state inactivation curve of *I*_Na(T)_ was shifted toward a depolarized potential, with no discernible modification in the slope factor of the curve; (iii) the decaying τ value of the cumulative inhibition of *I*_Na_ during PT depolarizing stimuli was increased by adding OM; (iv) the recovery τ value of *I*_Na_ following the slow inactivation of the current evoked by 10 s of maintained depolarization was overly diminished; (v) the strength of *I*_Na(W)_ activated by short ascending V_ramp_ became enhanced with the OM presence; (vi) OM had little or no effect on the magnitude of *I*_Ca,L_, while this compound slightly suppressed *I*_K(DR)_; (vii) in neuroblastoma Neuro-2a cells, the *I*_Na_ activated in response to a short depolarization pulse was also augmented by adding OM; and (viii) the molecular docking of OM to hNa_V_1.7 was performed based on the hypothesized formation of both hydrophobic contacts and hydrogen bonds between the ligand and the protein. Overall, this study presents initial evidence suggesting that OM can activate *I*_Na_ in GH_3_ and Neuro-2a cells, which appears to be unrelated to its impact on myosin motor.

The magnitude, gating properties, frequency dependence, and slow inactivation of *I*_Na_ caused by the OM presence led us to suggest that it preferentially binds to and stimulates the open/inactivated state (conformation) of the Na_V_ channel, thereby resulting in a stabilization in open conformation. The findings demonstrated herein also reflect that an increase in *I*_Na(T)_ and *I*_Na(L)_ may be part of the mechanisms by which OM produces a positive inotropic effect in the ventricular myocardium, assuming that OM may directly interact with Na_V_ channels to augment *I*_Na_ in heart cells occurring in vivo. Therefore, the observed perturbations of *I*_Na_ by OM are significant and may be linked to the neurological effects and proarrhythmic tendencies associated with OM use. These findings lend credence to the potential adverse effects of OM on ion-channel kinetics and its impact on overall cardiac function [20,26].

Our findings indicate that OM not only increased the amplitude of *I*_Na(T)_ but also caused a rightward shift of the quasi-steady-state inactivation curve of the current by approximately 11 mV. We did not observe any changes in the slope factor of the inactivation curve of the current in the presence of OM. The decaying τ value in the cumulative inhibition of *I*_Na_ during 1 s PT depolarizing stimuli also increased with the addition of OM. Additionally, the τ value in the recovery time course from the slow inactivation of *I*_Na_ following a 10 s depolarizing prepulse from −80 to −10 mV was reduced as cells were exposed to OM. Moreover, the strength of *I*_Na(W)_ in response to short ascending V_ramp_ was augmented by the addition of OM. It is reasonable to assume that the sensitivity of excitable cells, such as GH_3_ and Neuro-2a cells, to OM may depend on several factors, including the preexisting level of resting membrane potential, the firing rate of action potentials, and the concentration of OM used. However, the exact interplay between these factors and the action of OM in vivo on neurons or neuroendocrine cells remains to be determined. Therefore, caution should be exercised when extrapolating the observed effects of OM on GH_3_ or Neuro-2a cells to other cell types or physiological contexts.

The present observations highlight that the instantaneous *I*_Na(W)_ evoked by an abrupt ascending V_ramp_ can be enhanced by the addition of OM. While the overall steady-state *I*–*V* relationship of *I*_Na_ remained unaltered in the presence of OM, this compound had the potential to increase the strength of *I*_Na(W)_ and shift the inactivation curve of *I*_Na_ towards the depolarized direction. Thus, the modifications of *I*_Na(T)_, *I*_Na(L)_, and *I*_Na(W)_ by OM are potentially important and should not be underestimated. It is essential to exercise caution in attributing the action of OM on the functional activities of excitable cells to either the activation of myosin motor or the stimulation of ryanodine receptors, as reported in previous studies [1,2,4,6,9,26].

In our study, we did not observe any effect of OM on the magnitude of *I*_Ca,L_, although a slight inhibitory effect on *I*_K(DR)_ was detected in its presence. These findings are consistent with previous studies that showed little or no effect of OM on cytosolic Ca^2+^ transients [3,33]. However, other studies have reported that OM is capable of prolonging the duration of the contractile force or Ca^2+^ transients in isolated human atrium or heart cells, despite no change in the contractile force [34,35]. Therefore, further investigation is necessary to determine the extent to which OM-induced prolongation of the contractile duration of cardiac muscle is due to its preferential stimulation of *I*_Na(L)_ [15,16,17,18,19].

The maximal blood concentration of OM following intravenous or oral administration has been reported to reach approximately 800 ng/mL (equivalent to around 2 µM) [10]. In patients with impaired renal function, the concentration may be even higher [36]. Studies have shown that OM concentrations of 1 and 10 µM can increase contractility in single heart cells [33,37]. Therefore, the concentrations of OM used in this study to increase *I*_Na_ in GH_3_ and Neuro-2a cells are likely to be clinically or therapeutically achievable in healthy individuals or patients with heart failure [1,5,6,9,10,20].

The results of this study demonstrate that OM activates *I*_Na_ in GH_3_ and Neuro-2a cells and suggest that this effect may also extend to other types of electrically excitable cells, including heart cells. It is possible that OM interacts with other isoforms of Na_V_ channels, such as Na_V_1.5 channels, which are considered promising targets for improving cardiac function. Previous studies have shown that OM improves systolic function [1,2,6], partially through its action on Na_V_ channels at an as-yet-unidentified site. Therefore, the pharmacological or therapeutic actions of OM that are not related to its binding to myosine inside the cell may be partly due to its stimulation of Na_V_ channels, even though the detailed mechanism of OM’s action on the channels remains unclear.

## Data Availability

The original data are available upon reasonable request to the corresponding author.

## Abbreviations

EC_50_	concentration required for 50% stimulation
*I*–*V*	current versus voltage
*I*_Ca,L_	L-type Ca^2+^ current
*I*_K(DR)_	delayed-rectifier K^+^ current
Hys_(V)_	voltage-dependent hysteresis
*I*_Na_	voltage-gated Na^+^ current
*I*_Na(L)_	late Na^+^ current
*I*_Na(T)_	transient Na^+^ current
*I*_Na(W)_	window Na^+^ current
Na_V_ channel	voltage-gated Na^+^ channel
Nif	nifedipine
*I*–*V* relationship	current versus voltage relationship
OM	omecamtiv mecarbil (CK-1827452)
PT	pulse train
SEM	standard error of mean
τ	time constant
TEA	tetraethylammonium chloride
TTX	tetrodotoxin
V_ramp_	ramp voltage
